# Influence of Hot Plastic Deformation in γ and (γ + α) Area on the Structure and Mechanical Properties of High-Strength Low-Alloy (HSLA) Steel

**DOI:** 10.3390/ma9120971

**Published:** 2016-11-30

**Authors:** Jan Sas, Tibor Kvačkaj, Ondrej Milkovič, Michal Zemko

**Affiliations:** 1 Institute for Technical Physics, Karlsruhe Institute of Technology, 76344 Eggenstein-Leopoldshafen, Germany; 2 Institute of Materials, Technical University of Kosice, 04200 Kosice, Slovakia; tibor.kvackaj@tuke.sk; 3 Institute of Materials Research, Slovak Academy of Sciences, 04200 Kosice, Slovakia; omilkovic@saske.sk; 4 COMTES FHT a.s., Průmyslová 995, 33441 Dobřany, Czech Republic; mzemko@comtesfht.cz

**Keywords:** high-strength low-alloy (HSLA) steel, controlled rolling, mechanical properties, microstructure, dual-phase region

## Abstract

The main goal of this study was to develop a new processing technology for a high-strength low-alloy (HSLA) steel in order to maximize the mechanical properties attainable at its low alloy levels. Samples of the steel were processed using thermal deformation schedules carried out in single-phase (γ) and dual-phase (γ + α) regions. The samples were rolled at unconventional finishing temperatures, their final mechanical properties were measured, and their strength and plasticity behavior was analyzed. The resulting microstructures were observed using optical and transmission electron microscopy (TEM). They consisted of martensite, ferrite and (NbV)CN precipitates. The study also explored the process of ferrite formation and its influence on the mechanical properties of the material.

## 1. Introduction

Where strength must be improved while maintaining high formability, controlled rolling (CR) and controlled cooling (CC), also referred to as thermomechanical control processing (TMCP), are often used. Microalloyed high-strength low-alloy (HSLA) steels are low-carbon, low-alloy steels which contain micro additions of alloying elements, such as Nb, V or Ti [1,2]. HSLA steels with a multiphase microstructure have been extensively studied in the last two decades. These steels exhibit high strength, high formability, low-temperature toughness, resistance to hydrogen-induced cracking, resistance to fatigue, and good weldability, particularly if their carbon content is kept below 0.1 wt %. Several categories of HSLA steels are popular in specific applications, such as the construction of large ships, bridges, buildings, pressure vessels, tubes and pipelines, transport engineering, vehicles, etc. [3,4,5].

Grain refinement is achieved by controlling the rolling conditions: time, temperature, deformation, and cooling rate. It is one of the most effective strengthening mechanisms available for improving mechanical and fracture properties in steels at low temperatures. Its operation can be described by the Hall-Petch formula [6,7]. According to the authors of [8,9], the methods of grain refinement in steel can be divided into the following rolling and cooling procedures which involve reheating and normalized controlled rolling performed in the region of the spontaneous recrystallization of austenite:

(i). when the austenitic grain size is refined by repeating cycles which comprise deformation + recrystallization followed by phase transformation to ferrite (dγ ≈ 20–30 μm and dα ≈ 10–20 μm)
(ii). with deformation that continues to the region of the retarded recrystallization of austenite when austenite grains become elongated, followed by phase transformation to fine ferrite (Sv(gb + db) ≈ 50–500 mm^−1^, dγ,cor ≈ 20–30 μm, and dα ≈ 10–20 μm)
(iii). with deformation that continues to the region of the retarded recrystallization of austenite and the dual-phase region (γ + α), when austenite grains become elongated, and ferrite grains and subgrains form during deformation, followed by a phase transformation to very fine ferrite (Sv(gb + db) ≈ 700 mm^−1^, dγ,cor ≈ 4 μm, and dα ≈ 1 μm)

Therefore, it was necessary to investigate the effects of various controlled-rolling processes that involve deformation in the recrystallization region, in the γ non-recrystallization region, and in (γ + α) regions on the structure and properties of the present niobium- and vanadium-containing HSLA steel [6,7]. Previous investigations [10,11,12] studied separately either the influence of CR + CC conditions on microstructure evolution [10] or their impact on mechanical properties [11].

The present paper describes an investigation into a novel thermomechanical process related to the dual-phase region (γ + α), which involves arresting the microstructure evolution by water quenching immediately after deformation. Thanks to this arrangement, not only the formation of ferrite and the ferrite fraction can be studied but also the mechanical properties and their interrelationship as a function of thermal and deformation conditions can be explored.

## 2. Experimental Procedure

The experimental steel was produced by melting in an arc furnace with subsequent continuous casting. The resulting square billet was hot forged to a size of 200 mm × 200 mm. Table 1 gives its chemical composition measured by a Bruker Q4 TASMAN 130 spectroscope (Bruker Elemental GmbH, Kalkar, Germany).

**Figure 1:**
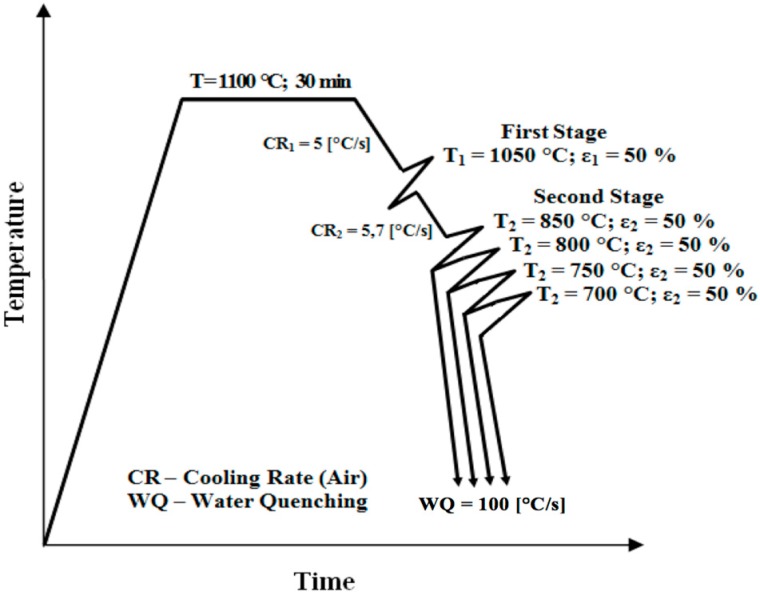
Experimental schedules.

The forged billets of 200 mm × 200 mm in cross-section were cut into experimental samples of 25 mm × 30 mm × 70 mm size. These were soaked at 1100 °C in a laboratory chamber furnace for 30 min to obtain homogeneous and equal recrystallized austenitic microstructures across all samples. They were then controlled-rolled in the experimental rolling mill DUO 210 according to schedules shown schematically in Figure 1.

## 3. Results and Discussion

### 3.1. Effect of Finish Rolling Temperature on the Final Microstructure

**Figure 2:**
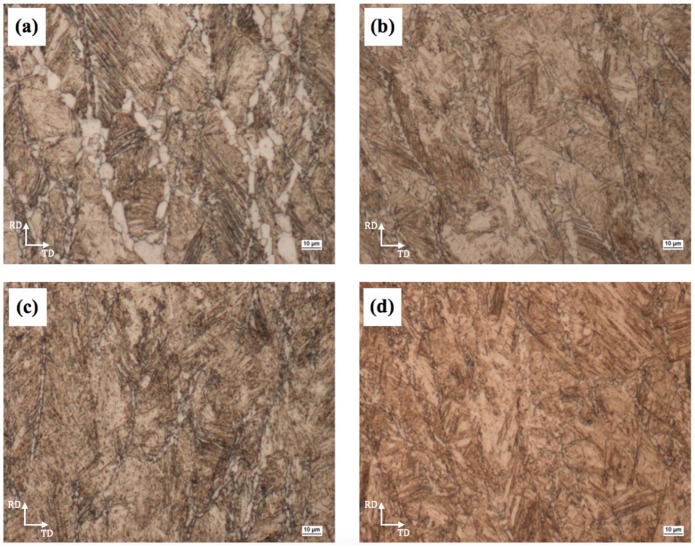
Effect of the FRT on final microstructures in specimens after controlled rolling with the final reduction of 50% (RD—rolling direction, TD—transverse direction): (**a**) 700 °C; (**b**) 750 °C; (**c**) 800 °C; (**d**) 850 °C.

Microstructures observed by a light microscope in the rolled and water-quenched specimens (Figure 2) consist predominantly of lath martensite which formed in pancaked prior austenite grains, and ferrite. Effects of controlled rolling and controlled cooling in the γ single-phase and (α + γ) two-phase regions of the finish rolling temperature (FRT) on the grain size and volume fraction of ferrite and on the resulting microstructure were studied.

**Figure 3:**
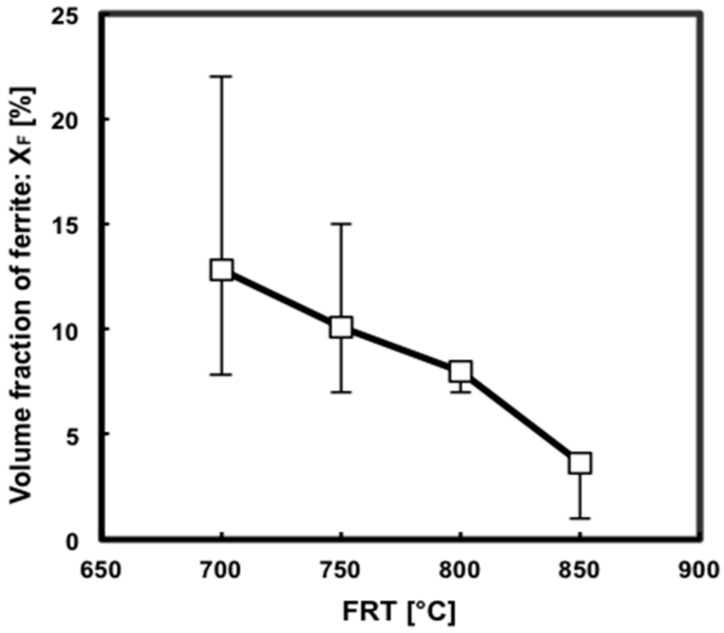
Variation in the volume fraction of ferrite with the finish rolling temperature.

The volume fraction of ferrite increased with the decreasing FRT, as evidenced by Figure 3. The ferrite grain size varied with the rolling temperature as well. Larger amounts of ferrite were found in those samples in which cooling began and deformation was imparted at lower temperatures, which thus accelerated the transformation process (Figure 3). In order to design a suitable reheating schedule, we calculated the A_r3_ phase transformation temperature of the steel before the hot-rolling experiment. For this calculation, the JMatPro simulation software (version 8.0, COMTES FHT a.s., Pilsen, Czech Republic) was used (approximate A_r3_ = 790 °C, as shown in Table 1).

The purpose of quenching was to preserve the ferrite that had formed by meta-dynamic transformation. Microstructure observation revealed that equiaxed ferrite and martensite were obtained, as shown in Figure 2. Therefore, the microstructure characterization of the quenched sample was then employed to study the deformation-induced ferrite transformation (DIFT). This mechanism involves the dynamic formation of nuclei and the transformation process being finalized statically. However, the microstructure that formed metadynamically is very easy to change by quenching because of the low stability of the deformed low-carbon austenite. From the viewpoint of thermodynamics, a prominent feature of DIFT that distinguishes it from static transformations without deformation is the added deformation energy which becomes the driving force of the transformation. This leads to an increase in the A_e3_ temperature or greatly accelerates transformation at temperatures between A_e3_ and A_r3_. The influence of the DIFT on ferrite nucleation was observed at temperatures higher than A_r3_, as shown in Figure 3. In terms of kinetics, the DIFT is a nucleation-dominated process. By contrast, continuous cooling transformation or isothermal transformation without deformation are grain growth–dominated processes [12,13,14,15].

**Figure 4:**
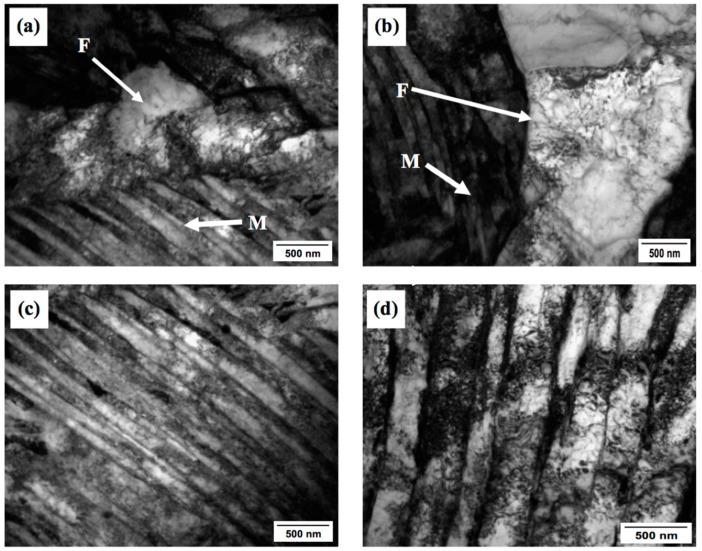
(**a**) Transmission electron microscopy (TEM) bright-field (BF) micrograph showing the ferritic microstructure (F) upon the FRT of 850 °C; (**b**) 700 °C; (**c**) Martensite lath structures (M) upon the FRT of 850 °C and (**d**) 700 °C.

Accelerated cooling after deformation and deformation of austenite below the recrystallization temperature typically lead to ferrite grain refinement [16]. The nucleation rate of ferrite is accelerated by: (i) bulges formed by local austenite grain boundary migration [6]; (ii) formation of subgrains near distorted austenite grain boundaries; and (iii) the strain energy of dislocations stored in deformed austenite [17]. The grain refinement process is governed by temperature, deformation and time. The ferrite grain size is also affected by deformation in the second stage of the sequence shown in Figure 1. Multiple authors [16,18] have reported that the ferrite grain size decreases with increasing rolling reductions. During rolling in the two-phase region, both austenite and ferrite grains are compressed. The austenite grains become elongated and provide larger surfaces for nucleation and phase transformation. Within the ferrite grains, dislocation cells form which contribute to grain refinement by the conversion of low-angle grain boundaries to high-angle boundaries. More detailed observations of microstructure variation with changing the finish rolling temperatures were carried out using transmission electron microscopy (TEM). Upon water quenching from various FRTs, the deformed austenite transformed predominantly to lath martensite and ferrite with high dislocation densities, as is shown in Figure 4a–d.

**Figure 5:**
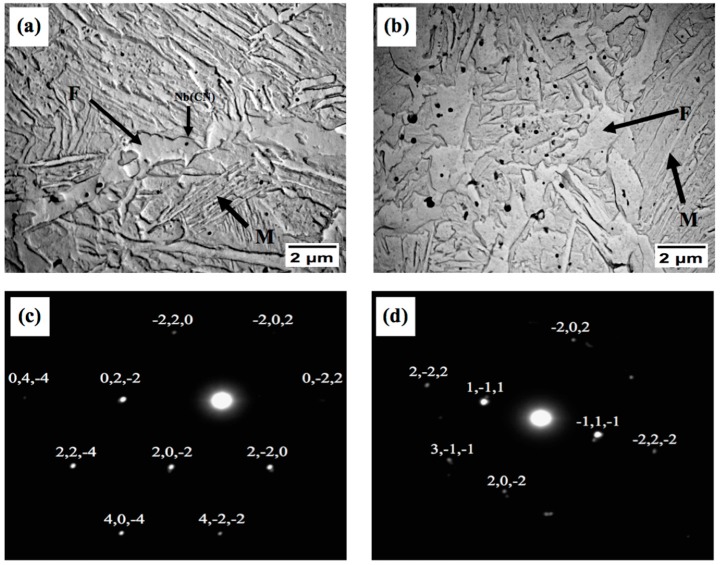
(**a**) TEM micrographs from carbon extraction replicas showing dark precipitates NbCN within ferrite and martensite obtained upon schedules with the FRT = 850 °C and (**b**) FRT = 700 °C; (**c**,**d**) SADPs of particles indicated by arrows in micrographs above; (**c**) FRT = 850 °C, (**d**) FRT = 700 °C.

The transformation within the still-warm austenite may produce dislocations within martensite laths due to accommodation of the volume change–induced strain. The dependence of the dislocation density within ferrite and the thickness of martensite laths on the FRT can be seen in Figure 4. It is evident that the width of the martensite laths decreases and the dislocation density increases with the increasing FRT (Figure 4c,d). The reason can be the smaller volume fraction of ferrite that exists at higher FRTs because the strain energy introduced during rolling becomes absorbed by this ferrite constituent. At higher FRTs, the resulting microstructure contains less ferrite and more martensite laths, which leads to a large strain and higher dislocation densities in ferrite, and larger strain within martensite laths. Figure 5a,b show TEM micrographs from carbon replicas with dispersed precipitates in the form of globular dark particles. The selected area diffraction pattern (SADP) with indices shown in Figure 5c,d confirms the presence of NbCN precipitates after finish rolling at 850 °C and 700 °C. Their density increases with the decreasing FRT.

The particles can be divided according to their size into the following groups. The first group is (i) *d* > 100 nm; these were identified using diffraction as NbCN particles which do not dissolve at the soaking temperature of 1100 °C. They were found predominantly within ferrite grains. The second group is (ii) *d* < 100 nm; these particles were identified as NbCN as well. An additional minor proportion of (NbV)CN precipitates can be expected to be present. These may be the grain boundary precipitates, although their vanadium content has not been proved by diffraction [19,20].

### 3.2. Effect of Finish Rolling Temperature on Mechanical Properties

**Figure 6:**
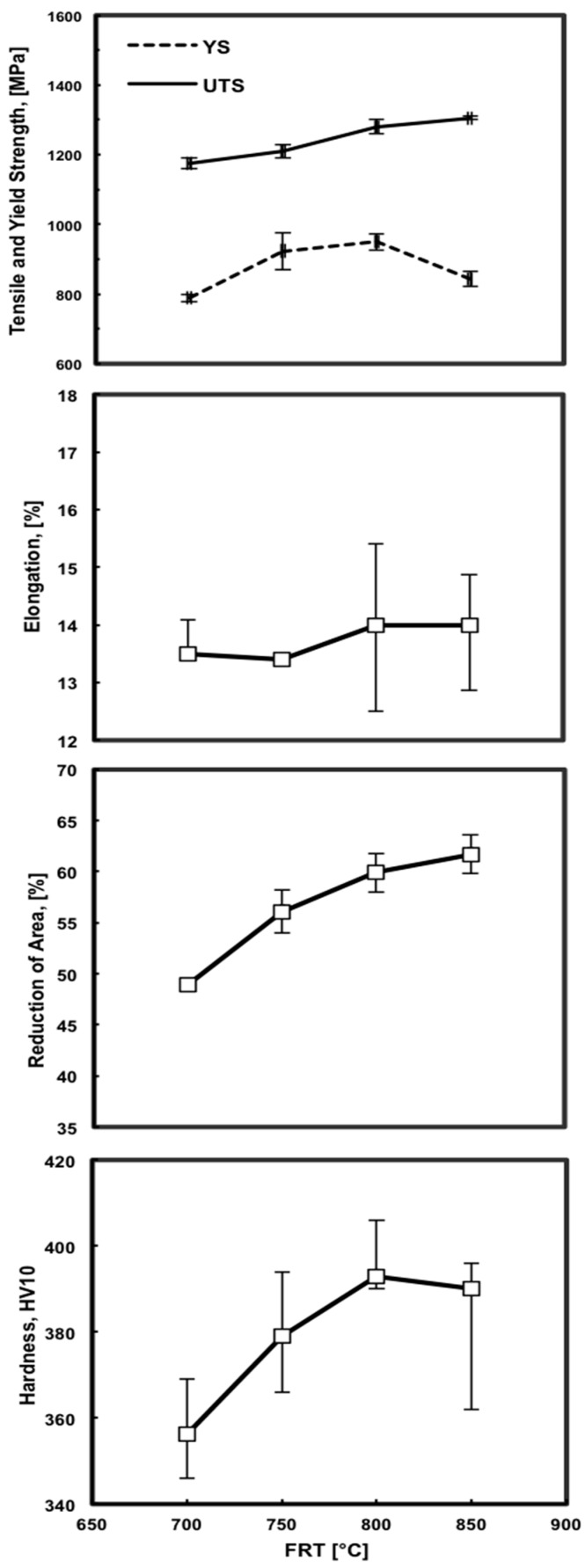
Variation in yield strength (YS), ultimate tensile strength (UTS), elongation, reduction of area and hardness with the finish rolling temperature.

Figure 6 shows the variation in yield strength (YS), ultimate tensile strength (UTS), elongation, reduction of area (RA) and hardness of the water-quenched steel with the FRT. The lowest values of YS (789 MPa), UTS (1175 MPa), elongation (13%), RA (49%) and hardness (356 HV10) were obtained at the lowest FRT. It appears that strength characteristics (YS, UTS) depend more strongly on the decrease in the finish rolling temperature than on precipitation strengthening. In this context, it can be mentioned that the YS is more sensitive than the UTS at the FRT of 850 °C. It is important to note that the YS keeps increasing with the FRT up to 800 °C due to complete NbCN precipitation and incipient the vanadium carbide (VC) precipitation from the solid solution. The fall-off in the YS curve and the point of inflection in the UTS curve may correlate with deformation-induced phase transformation and with an increase in the volume fraction of ferrite which had a greater impact on the strength than precipitation strengthening. All elongation values are close to the average value of approximately 14%. The effect of the FRT on elongation was minimal. The decrease in RA with the FRT, as shown in Figure 6, is related to two concurrently increasing but competing factors: the volume fraction of ferrite and the amount of precipitation strengthening. The decrease in hardness with the FRT is due to the growing volume fraction of softer ferrite at the expense of the volume of martensite.

## 4. Conclusions

The effects of the finish rolling temperature on the microstructure and mechanical properties of C-Mn-Nb-V steel have been investigated. The results indicate that it is possible to improve the strength properties of this microalloyed steel by a controlled rolling and controlled cooling sequence in γ single-phase and (γ + α) two-phase regions. The final microstructures of the samples were mixtures of ferrite and lath martensite. During the transition from the γ region to the (γ + α) region, the fraction of ferrite increases in the interval X_F_ (0.8,18.4) [%] with the decreasing FRT. It has been shown that their volume fractions correlate with the values of the tensile and yield strengths, elongation, reduction of area and hardness. Both constituents, ferrite and martensite, exhibited high dislocation densities. TEM analysis revealed complex precipitates of microalloying elements (NbV)CN. The highest yield strength (822–864 MPa), ultimate tensile strength (1305–1310 MPa), elongation (13%–15%), reduction of area (60%–64%), and hardness (362–396 HV10) values in the water-quenched steel samples were achieved with the schedule that involved the finish rolling temperature (FRT) of 850 °C. An increase in strength without any substantial changes in elongation is achieved by the refinement of martensite laths and by the decreasing volume fraction of ferrite as the finish rolling temperature increases.

## Figures and Tables

**Table 1:** Chemical composition (weight percent: wt %) of the investigated steel.

C	Mn	Si	P	S	Cu	Ni	Cr	Ti	Nb	V	CE *
0.12	1.54	0.12	0.004	0.001	0.12	0.09	0.15	≤0.01	0.048	0.18	0.48

* Carbon equivalent (CE) = C + (Mn + Si)/6 + (Ni + Cu)/15 + (Cr + Mo + V)/5 = 0.48; **A_r3_** = 790 °C.
